# Gamma and Theta/Alpha-Band Oscillations in the Electroencephalogram Distinguish the Content of Inner Speech

**DOI:** 10.1523/ENEURO.0297-24.2025

**Published:** 2025-02-06

**Authors:** Thomas J. Whitford, Kevin M. Spencer, Marianthe Godwin, Yoji Hirano, Lawrence Kin-hei Chung, Wadim Vodovozov, Oren Griffiths, Anthony W. F. Harris, Mike E. Le Pelley, Bradley N. Jack

**Affiliations:** ^1^School of Psychology, University of New South Wales (UNSW Sydney), Sydney, New South Wales 2052, Australia; ^2^Brain Dynamics Centre, Westmead Institute for Medical Research, Sydney, New South Wales 2145, Australia; ^3^Research Service, VA Boston Healthcare System, and Department of Psychiatry, Harvard Medical School, Boston, Massachusetts 02130; ^4^Department of Psychiatry, Division of Clinical Neuroscience, Faculty of Medicine, University of Miyazaki, Miyazaki 889-2192, Japan; ^5^Department of Psychology, The Chinese University of Hong Kong, Hong Kong 999077, Hong Kong SAR, China; ^6^Department of Psychiatry, Zucker Hillside Hospital, Glen Oaks, New York 11004; ^7^School of Psychology, University of Newcastle, Newcastle, New South Wales 2308, Australia; ^8^Speciality of Psychiatry, Sydney Medical School, University of Sydney, Sydney, New South Wales 2006, Australia; ^9^Research School of Psychology, Australian National University, Canberra 0200, Australian Capital Territory, Australia

**Keywords:** auditory evoked potential, EEG, gamma, inner speech, N1, prediction error

## Abstract

Inner speech refers to the silent production of language in one’s mind. As a purely mental action without obvious physical manifestations, inner speech has been notoriously difficult to quantify. To address this issue, the present study repurposed the phenomenon of speaking-induced suppression, wherein overt speech has been consistently shown to elicit reduced auditory evoked potentials compared with externally generated speech, as well as changes in oscillatory activity in gamma and theta frequency bands. Given the functional similarities between inner and overt speech, we used an established experimental protocol to investigate whether similar metrics could be used to distinguish the content of inner speech. Healthy participants (*n* = 129) produced an inner syllable at a precisely specified time. An audible syllable was concurrently presented which either matched or mismatched the content of the inner syllable. The results revealed that Match and Mismatch conditions could be differentiated on the basis of their evoked oscillations in the gamma, theta, and alpha bands. Notably, there was a gamma-band oscillation in the vicinity of the P2 that differed between the Match and Mismatch conditions, suggesting that “late” gamma-band activity may index consciously perceived expectancy violations, or cognitive prediction errors. Regarding the auditory evoked potentials, the N1 component was suppressed in the Match condition while the P2 component was suppressed in the Mismatch condition, replicating previous findings. This study provides support for the existence of “inner speaking-induced suppression”, and demonstrates that inner syllables can be differentiated based on their influence on the electroencephalographic activity elicited by simultaneously-presented audible syllables.

## Significance Statement

Inner speech refers to the silent production of language in one’s mind. As a purely mental action without obvious physical manifestations, inner speech has been notoriously difficult to quantify empirically. The results of the present study demonstrate that it is possible to distinguish between two syllables produced in inner speech on the basis of their influence on the electroencephalographic activity elicited by simultaneously-presented audible syllables; specifically, the evoked oscillations in the gamma, theta, and alpha frequency bands, and the N1- and P2-components of the auditory evoked potential. The ability to determine the content of a person’s inner speech on the basis of a non-invasive biometric signal could have significant commercial, industrial, and clinical applications.

## Introduction

Inner speech refers to the silent production of language in one's mind (Alderson-Day and Fernyhough, 2015). It is a ubiquitous human activity that is believed to play a role in numerous mental functions including silent reading, memory, logical reasoning, and cognitive control. A prominent theory argues that inner speech arises developmentally from overt speech, and ultimately reflects an internalized version of overt speech in which the articulator organs, such as the mouth, tongue, and larynx, do not move (Jones and Fernyhough, 2007).

As a purely mental action with no obvious physical manifestations, inner speech has been notoriously difficult to study empirically. We (Whitford et al., 2017; Jack et al., 2019), and others (Tian and Poeppel, 2015; Tian et al., 2018), have previously shown that it is possible to differentiate between syllables produced in inner speech (“inner syllables”) on the basis of their influence of the auditory evoked potential elicited by a simultaneously presented audible sound (“audible syllables”). Specifically, when participants were asked to produce an inner syllable at a precisely designated time concurrent with the presentation of an audible syllable, the amplitude of the N1 component of the auditory evoked potential was reduced if, but only if, the content of the inner syllable matched the content of the audible syllable. Conversely, the amplitude of the P2 component was found to be reduced when the content of the inner syllable mismatched the content of the concurrently presented audible syllable.

These results are consistent with the phenomenon of speaking-induced suppression (SIS) in the context of overt speech (Whitford, 2019). SIS refers to the finding that the sounds generated by willed vocalizations elicit a smaller neurophysiological response—such as smaller N1 and/or P2 components (Sato and Shiller, 2018)—than do externally generated sounds that are physically identical (Niziolek et al., 2013; Gonzalez et al., 2024). SIS is believed to result from the activity of a corollary discharge (Crapse and Sommer, 2008; Schneider et al., 2014), which is a movement-related neural signal that is transmitted to sensory areas and which contains predictions about sensory consequences of self-generated movements. Corollary discharges are believed to play a role in predicting the sensory properties of overt speech, such as its content, temporal onset, and auditory properties such as pitch and loudness. Given the notable similarities between the phenomenon of SIS and our inner speech findings, we and others have previously suggested that inner speech may also elicit a corollary discharge that contains information about the properties of that inner speech, such as its content, onset, and maybe even equivalents of auditory properties: e.g., “inner pitch” and “inner loudness” (Scott, 2013; Whitford et al., 2017; Tian et al., 2018; Jack et al., 2019; Whitford, 2019; Li et al., 2020; Zheng et al., 2022; Chung et al., 2023). This suggestion is consistent with the idea that inner speech is ultimately a special case of overt speech in which the articulator organs do not move.

Overt speech has been shown to elicit synchronous neural oscillations in the gamma frequency band (30–100 Hz; Crone et al., 2001; Ford et al., 2005; Fukuda et al., 2010; Chen et al., 2011; Basilakos et al., 2017; Tsunada and Eliades, 2020). Consistent with the conceptualization of inner speech as being related to overt speech, there is also some evidence that gamma-band oscillations are associated with the production of inner speech (Moon et al., 2022). While gamma-band oscillations have been associated with numerous cognitive functions in the literature—including feature binding, attention, memory, and motor coordination; see Uhlhaas et al. (2008) for a review—of particular relevance to the current paper is its suggested role in the transmission of a corollary discharge (Moon et al., 2022; Moon and Chau, 2023). There are at least three lines of evidence to support this idea: firstly, evoked power in the gamma frequency band is reduced during the production of overt speech compared with passive listening (Baess et al., 2009); secondly, evoked gamma power is higher to unpredicted (i.e., distorted) auditory feedback than to predicted (i.e., undistorted) feedback during speech production (Behroozmand et al., 2016); thirdly, studies investigating gamma-band coherence between the frontal and temporal lobes have found coherence to be higher during overt speech production compared with listening (Chen et al., 2011) and to predicted versus unpredicted auditory feedback (Ford et al., 2005). Given that these studies all compared the spectral activity elicited by self-generated and externally generated speech sounds, their results collectively suggest that oscillations in the gamma-band may be involved in distinguishing between “self” and “world” and thus may be related to corollary discharges, given that corollary discharges are known to be involved in this distinction.

It is important to note that gamma is not the only frequency band that has been associated with speech production. For example, there is strong evidence for the phenomenon of “speech-to-brain” entrainment, in which the acoustic features of speech are reflected in neural oscillations at equivalent frequencies, which roughly corresponds to the theta band (4–8 Hz) for syllable perception (Luo and Poeppel, 2007; Ding et al., 2016; Keitel et al., 2018). Furthermore, there is also evidence that the production of inner speech is associated with changes in oscillatory activity in the theta band. For example, Yao et al. (2021) found that reading “direct” quotes (e.g., Gareth bellowed “It looks like there is nobody here!”) was associated with increased phase-locking in the theta frequency band compared with when reading “indirect” quotes (e.g., Gareth bellowed that there looked like nobody was there). Given that reading direct quotes is believed to induce more vivid inner speech than reading indirect quotes (Yao et al., 2011; Yao and Scheepers, 2015), this result suggests that theta-band activity may be associated with the vividness of inner speech. Given this, the present study will also investigate the impact of inner speech production on evoked power at lower, sub-gamma frequency bands (5–30 Hz).

The first aim of the present study was to investigate, in a large sample (*n* = 129) of psychologically healthy participants, whether the content of an inner syllable can be determined on the basis of its influence on the level of gamma-band oscillatory activity elicited by a simultaneously presented audible syllable. This will shed light on whether the corollary discharge that we, and others, have suggested is associated with inner speech is associated with gamma-band activity. The second aim was to investigate whether inner speech production was also associated with activity in lower (i.e., sub-gamma) frequency bands, particularly theta, and whether these changes were similarly sensitive to the content of inner speech.

## Materials and Methods

### Participants

One hundred and thirty-three individuals were recruited for the study; however, four participants were excluded for having excessive artifact in their electroencephalogram (EEG) recording (see below, EEG processing and analysis, for further details). This left a final sample of 129 participants; mean age was 20.3 years (SD = 2.8), 78 were female (60%), and 116 (90%) were right-handed. All participants were undergraduate students from the University of New South Wales (UNSW) who participated for course credit. The study was approved by the UNSW Human Research Ethics Advisory Panel (Psychology).

### Apparatus, stimuli, and procedure

Participants were seated in a quiet, dimly lit room, ∼60 cm from a computer monitor (BenQ XL2420T, 1,920 × 1,080 pixels; diagonal length of monitor, 64 cm) and were fitted with headphones (AKG K77 Perception). Participants also wore a face mask for the purposes of COVID control. Stimulus presentation was controlled by custom-made MATLAB scripts using the Psychophysics Toolbox (Brainard, 1997; Kleiner et al., 2007).

**Figure 1.**
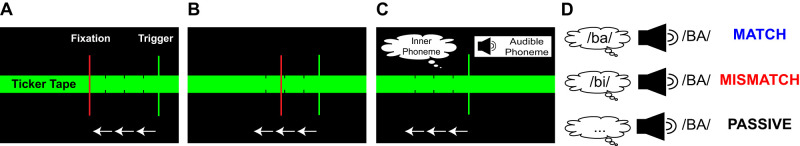
A schematic of the experimental protocol. Participants were initially instructed to fixate their eyes on the central red fixation line, which was embedded in the thick green horizontal bar (the “tickertape”; see panel ***A***). After a 1 s delay, the green trigger line, which was initially presented near the right side of the screen, and visible in participants’ peripheral vision, began to move slowly across the screen in a leftward direction at a speed of 6.5° of visual angle per second (see panel ***B***), such that after ∼3.75 s, the green trigger line overlapped with the red fixation line. At this exact time, dubbed the “sound time,” two events occurred simultaneously. Firstly, participants were asked to imagine themselves moving their mouth and making a predefined syllable in their inner speech. Depending on the block, participants were asked to produce the inner syllable /ba/, the inner syllable /bi/, or no inner syllable. Secondly, an audible syllable was delivered to participants’ headphones; the audible syllable was of a male speaker producing the syllable /BA/ or /BI/. In Match trials (panel ***D***, top row, blue), the content of the inner syllable was congruent with the content of the audible syllable (e.g., inner syllable, /ba/; audible syllable, /BA/). In Mismatch trials (panel ***D***, middle row, red), the content of the inner syllable was incongruent with the content of the audible syllable (e.g., inner syllable, /bi/; audible syllable, /BA/). In Passive trials (panel ***D***, bottom row, black), the participant did not produce an inner syllable (e.g., inner syllable, none; audible syllable, /BA/). Following the “sound time,” the trigger line continued to move leftward past the fixation line for an additional 1 s. The participant was then asked to rate how successfully they were able to follow the instructions on the trial, on a 5-point Likert scale, ranging from 1 (“not at all successful”) to 5 (“completely successful”).

The experimental protocol was based on the task used by Whitford et al. (2017). On every experimental trial, participants saw an animation on the screen (Figure 1). The animation consisted of a thick horizontal green line in the center of the screen (the “tickertape”), a thin vertical red line in the center of the screen (the “fixation line”), and a thin vertical green line which started near the right side of the screen (the “trigger line”). Participants were instructed to keep their gaze fixated on the fixation line (which remained stationary) for the duration of the trial. After a 1 s delay, the tickertape, and its embedded trigger line, began to move slowly leftward across the screen, at a speed of 6.5°/s, so that after 3.75 s the green trigger line intersected with the red fixation line—this was the “sound time.” At the “sound time,” two events occurred: (1) participants were instructed to produce a specific syllable in inner speech (an inner syllable), and (2) an audible syllable was presented to the participant's headphones of a male speaker producing either the syllable /BA/ or the syllable /BI/. The experimental manipulation was the content of the inner syllable participants were asked to produce at the “sound time.” There were three different types of trial blocks: “imagine /BA/” blocks in which the participant was asked to produce the inner syllable /BA/, “imagine /BI/” blocks in which they were asked to produce the inner syllable /BI/, and “passive” blocks in which they were asked not to produce an inner syllable. After the “sound time,” the tickertape continued moving for an additional 1 s, before the participant was asked to rate their performance on the trial (i.e., how well they were able to produce the inner syllable, or passively listen), on a 5-point Likert scale, with scores ranging from 1 (“very bad”) to 5 (“very good”). We used these ratings to exclude trials in which participants self-reported as not successfully performing the task. Each block consisted of 24 trials; the audible syllable /BA/ was presented on half of the trials, while the audible syllable /BI/ was presented on the other half, in random order. There were four block repeats for each of the three trial types (i.e., Imagine /BA/ blocks, Imagine /BI/ blocks, Passive blocks), and the order of the blocks was randomized.

#### EEG acquisition

EEG data was recorded with a BioSemi ActiveTwo system using 64 Ag/AgCl active electrodes placed according to the extended 10–20 system (FP1, FPz, FP2, AF7, AF3, AFz, AF4, AF8, F7, F5, F3, F1, Fz, F2, F4, F6, F8, FT7, FC5, FC3, FC1, FCz, FC2, FC4, FC6, FT8, T7, C5, C3, C1, Cz, C2, C4, C6, T8, TP7, CP5, CP3, CP1, CPz, CP2, CP4, CP6, TP8, P9, P7, P5, P3, P1, Pz, P2, P4, P6, P8, P10, PO7, PO3, POz, PO4, PO8, O1, Oz, O2, Iz). External electrodes were also placed on the left and right mastoid bones (for the purposes of offline re-referencing, see below, EEG processing and analysis), the outer canthus of the left and right eye (for the horizontal electrooculogram), and below the left eye (for the vertical electrooculogram, referenced to electrode Fp1). The sampling rate of the EEG was 2,048 Hz.

### EEG processing and analysis

#### ERP data

The EEG preprocessing and ERP analysis was performed in BrainVision Analyzer (version 2.2, Brain Products). Local COVID restrictions meant that participants were required to wear face masks, which made it impractical to place an electrode on the nose. The data were instead rereferenced to the average of the mastoid electrodes, consistent with our procedure in Chung et al. (2023). Data were bandpass filtered from 0.1 to 100 Hz using a phase-shift free Butterworth filter (order 2), with a 50 Hz notch filter to minimize mains artifact. The filtered data were separated into 600 ms epochs (200 ms prior to sound onset, 400 ms postonset), corrected for eye movements using the technique described in Gratton et al. (1983). We also used ICA to identify and remove any headphone-related muscle artifact that occurred in the first 30 ms following on the onset of the audible syllable (Makeig et al., 1995). Any epoch with a signal exceeding a peak-to-peak amplitude of 200 µV were defined as unusable and excluded. To ensure data quality, epochs were also classified as unusable if the participant rated their success on the trials as <4 out of 5. The remaining usable epochs were included in the analysis; there was an average of 85.2 (SD = 14.6) usable trials in the Match condition (of a max. possible 96), 72.6 (SD = 23.2) usable trials in the Mismatch condition, and 81.7 (SD = 16.3) usable trials in the Passive condition. The remaining epochs were baseline corrected to the mean voltage of the interval from −200 to 0 ms and used to generate each participant's average ERP for the Match, Mismatch, and Passive conditions.

### Time–frequency data

For the gamma-band analysis, the Morlet complex wavelet transformation was applied to the 30–100 Hz frequency range of each participant's average waveform from the Match, Mismatch, and Passive conditions. There were 20 logarithmically spaced frequency steps with wavelet center frequencies at: 30, 31.963, 34.053, 36.281, 38.655, 41.183, 43.877, 46.748, 49.806, 53.064, 56.535, 60.234, 64.174, 68.372, 72.845, 77.610, 82.688, 88.097, 93.860, and 100 Hz. The Morlet parameter was set to 5. The output value was spectral power, in units of µV^2^. A decibel (dB) transform was applied to normalize power [10 × log 10 (power/baseline)]. The decibel transform removes scale differences between individuals, time points, and frequencies, rendering them more statistically comparable (Roach and Mathalon, 2008; Munneke et al., 2015). Baseline correction was applied per frequency band in the time window from −150 to −50 ms relative to the onset of the audible syllable. The epochs were clipped from −150 to 300 ms in order to remove edge artifacts.

For the analysis of the lower-frequency bands, the raw data were first reprocessed (Butterworth filter, 0.1–50 Hz plus 50 Hz notch) and resegmented into −600 to 800 ms epochs in order to account for the longer wavelets required for the analysis of the lower frequencies. The procedure for ocular correction, artifact rejection, and ICA was identical as for the gamma-band analysis. The Morlet complex wavelet transformation was applied to the 5–30 Hz frequency range, for each participant's average waveform, for each of the three conditions. There were 20 logarithmically spaced frequency steps with wavelet center frequencies at 5, 5.494, 6.038, 6.635, 7.291, 8.012, 8.804, 9.675, 10.632, 11.683, 12.839, 14.108, 15.504, 17.037, 18.722, 20.573, 22.608, 24.843, 27.300, and 30 Hz. The Morlet parameter was set to 5. A decibel (dB) transform was again applied to normalize power, and baseline correction was applied per frequency band in the time window −200 to 0 ms, which corresponded to a full cycle of the lowest frequency analyzed (5 Hz). The epochs were clipped from −200 to 600 ms in order to remove edge artifacts.

### Experimental design

As per the design of Whitford et al. (2017), the data were parsed into three distinct trial types, which were analyzed as separate conditions: Match trials, Mismatch trials, and Passive trials. Match trials were trials in which the inner syllable matched the content of the audible syllable (i.e., inner syllable = /BA/, audible syllable = /BA/ or inner syllable = /BI/, audible syllable = /BI/). Mismatch trials were trials in which the inner syllable did not match the content of the audible syllable (i.e., inner syllable = /BA/, audible syllable = /BI/, or inner syllable = /BI/, audible syllable = /BA/). Passive trials were trials in which participants did not produce an inner syllable (i.e., inner syllable = none, audible syllable = /BA/ or inner syllable = none, audible syllable = /BI/).

### Statistical analysis

#### ERP data

The ERP data were analyzed using a repeated-measures ANOVA, with two factors: Condition (three levels: Match, Mismatch, and Passive) and Electrode (nine levels, centered on the electrode for which the component-of-interest was maximal in the collapsed localizer waveform (Luck and Gaspelin, 2017); the maximal electrode was FCz for the N1 component (thus the electrodes-of-interest for the N1 analyses were Fz, FCz, Cz, F1, F2, FC1, FC2, C1, C2) and Cz for the P2 component (thus the electrodes-of-interest for the P2 analysis were FCz, Cz, CPz, FC1, FC2, C1, C2, CP1, CP2). There were two dependent variables, namely, the amplitude of the N1 and P2 components of the auditory evoked potential. As per the protocol of Whitford et al. (2017), the amplitude of the N1 component was quantified by picking the N1 peak on each participant's average ERP (the most negative local minimum in the time window 25–175 ms post-audible-syllable) for the Match, Mismatch, and Passive conditions separately, while the P2 component was quantified with a time window-based approach as the average voltage in the window 150–190 ms post-audible-syllable. In the case of a main effect of Condition, contrasts were used to unpack the simple effects. If the assumption of sphericity was violated, the Greenhouse–Geisser correction was applied. The ERP data were analyzed with SPSS Statistics 29 (IBM).

#### Time–frequency data

The statistical analysis for the time–frequency data was conducted in BESA Statistics 2.1 (BESA) which uses the cluster-based permutation testing algorithm developed by Maris and Oostenveld (2007). The procedure was identical for the analysis of the gamma and lower-frequency bands. Spectral power (µV^2^) for the 20 frequency bands was exported for all 12 central electrodes analyzed in the ERP analysis (viz., Fz, FCz, Cz, CPz, F1, F2, FC1, FC2, C1, C2, CP1, CP2) and compared between the Match, Mismatch, and Passive conditions. Nonparametric permutation testing was performed on the basis of the paired-samples *t* test (two-tailed; Maris and Oostenveld, 2007). The neighbor distance was set to the default setting of 4 cm, the cluster alpha level was set to 0.05, and 10,000 permutations were used. The output is corrected for multiple comparisons as only those clusters which have a cluster value (calculated as the sum of the *t* values of all time–frequency points in the cluster) that exceeds 95% of all clusters generated by a random permutation of the data are considered statistically significant.

## Results

### ERP data

**Figure 2.**
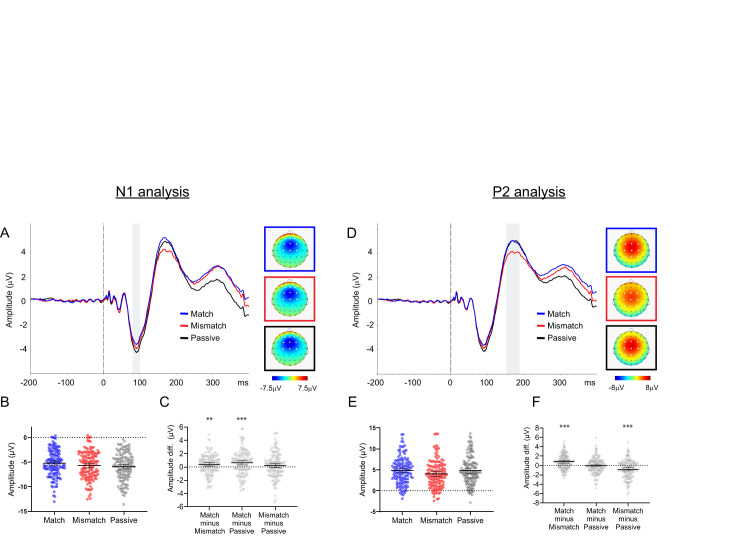
The results of the ERP analyses for the *n* = 129 participants. The left panels (panels ***A–C***) show the results of the analysis of the N1 component. Panel ***A*** shows the waveforms for the three conditions for the N1 analysis, time locked to the onset of the audible syllable. The waveforms are shown collapsed across the nine central electrodes at which N1 was maximal (FCz, FC1, FC2, Cz, C1, C2, Fz, F1, F2). The voltage maps for the N1 component are plotted for each condition as the average voltage in the time window 80–100 ms, represented by the gray vertical bar in Panel ***A***. Panel ***B*** shows scatterplots depicting the amplitude of the N1 component (calculated with a peak-picking approach) for each condition. Each dot represents a single participant's raw score, and the bars represent the mean and 95% CI. Panel ***C*** shows scatterplots depicting the within-subjects difference scores on N1 amplitude for the three contrasts-of-interest: namely, Match minus Mismatch, Match minus Passive, and Mismatch minus Passive. Each dot represents a single participant's difference score, and the bars represent the mean and 95% CI. The statistical significance of the between-condition contrasts are denoted with asterisks: *p < 0.05, **p < 0.01, ***p < 0.001. Note that as the N1 component has a negative voltage, a positive difference score for the “Match minus Passive” contrast is the same direction as the traditional “Self minus External” N1 suppression effect. The right panels show corresponding data for the analysis of the P2 component over the time window 150–190 ms (shown by the gray vertical bar in panel ***D***), collapsed over the 9 central electrodes at which P2 was maximal (CPz, CP1, CP2, FCz, FC1, FC2, Cz, C1, C2), which were slightly posterior relative to the N1 analysis. Voltage maps and data in panels ***E*** and ***F*** represent average voltage over the P2 time window. The statistical significance of the between-condition contrasts are denoted with asterisks: *p < 0.05, **p < 0.01, ***p < 0.001.

The auditory evoked potentials for the three conditions (Match, Mismatch, Passive), time locked to the onset of the audible syllable, are shown in Figure 2. Panels *A*–*C* show the analysis of the N1 component, while panels *D*–*F* show the analyses of the P2 component.

#### N1

The N1 peak was identified on each individual participant's average waveform for each of the three conditions. Figure 2*A* shows the auditory evoked potentials and voltage maps for the three conditions (Match, Mismatch, and Passive), collapsed across the nine electrodes-of-interest. Figure 2*B* shows the N1 peak amplitude for each participant for each of the three conditions. Finally, as this was a repeated-measures design, Figure 2*C* shows the within-subject difference scores between conditions for the N1 peak amplitude.

ANOVA revealed a main effect of Condition on the amplitude of the N1 peak, *F*_(2,256)_ = 8.775, *p* < 0.001, *η_p_*^2^ = 0.064. Analysis of simple effects revealed that the N1 amplitude in the Match condition was significantly smaller than both the Mismatch condition (*t*_(128)_ = 3.09, *p* = 0.002, 95% CI = [0.141, 0.645]) and the Passive condition (*t*_(128)_ = 3.94, *p* < 0.001, 95% CI [0.315, 0.950). The difference between the Mismatch and Passive conditions was not statistically significant (*t*_(128)_ = 1.44, *p* = 0.154, 95% CI = [−0.09, 0.569]).

#### P2

The P2 component was identified on the grand average waveform and analyzed with a time window-based approach within the window 150–190 ms poststimulus. Figure 2*D* shows the auditory evoked potentials and voltage maps for the three conditions, collapsed across the nine electrodes-of-interest. Figure 2*E* shows the P2 amplitude (average voltage within the P2 time window) for each participant for each of the three conditions. Figure 2*F* shows the within-subject difference scores between conditions for the P2 amplitude.

ANOVA revealed a main effect of Condition on the amplitude of the P2, *F*_(2,256)_ = 17.072, *p* < 0.001, *η_p_*^2^ = 0.118 (Greenhouse–Geisser corrected). Analysis of the simple effects revealed that the Mismatch condition had a significantly smaller P2 amplitude than both the Match condition (*t*_(128)_ = 5.93, *p* < 0.001, 95% CI = [0.527–1.055]) and the Passive condition (*t*_(128)_ = 4.30, *p* < 0.001, 95% CI = [0.430, 1.162]). There was no significant difference in P2 amplitude between the Match and Passive conditions (*t*_(128)_ = 0.03, *p* = 0.974, 95% CI [−0.297, 0.287].

### Time–frequency data

#### Gamma-band activity (30–100 Hz)

**Figure 3.**
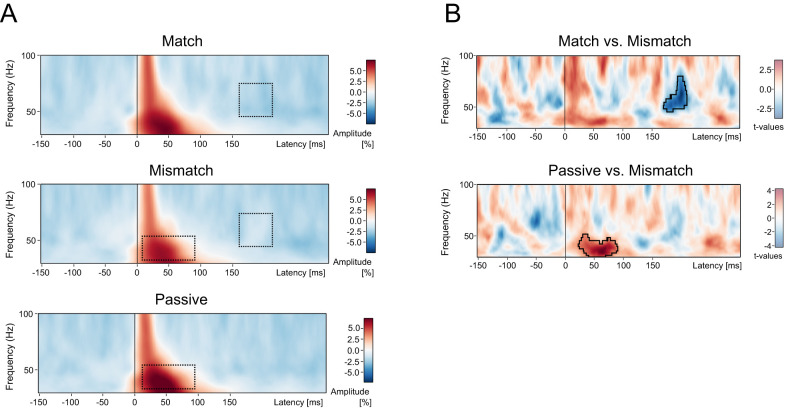
The results of the time–frequency analyses of the gamma-band activity (30–100 Hz) for the *n* = 129 participants. Panel ***A*** shows the spectral power from 30 to 100 Hz for each of the three conditions: Match (top row), Mismatch (middle row), and Passive (bottom row). The time–frequency plots are shown collapsed across the 12 electrodes-of-interest (i.e., Cz, C1, C2, FCz, FC1, FC2, CPz, CP1, CP2, Fz, F1, F2), time locked to the onset of the audible syllable, and baseline corrected using a decibel transform in the window −150 to 50 ms. The units are the percent change in amplitude relative to the baseline period. The results of the statistical contrasts are shown in panel ***B***; the units are *t* values. Nonparametric permutation testing (10,000 permutations) was performed on the basis of the paired-samples *t* test (2-tailed). Only those clusters with a cluster value (sum of *t* values of all time–frequency points in the cluster) exceeding 95% of all clusters generated by the permutation procedure were considered statistically significant. Panel ***B*** (top row) shows the single significant cluster of relative amplitude difference between the Match and Mismatch conditions. This cluster, outlined exactly in panel ***B*** and as a dotted schematic box in panel ***A***, extended from ∼45 to 80 Hz, was apparent from ∼170 to 250 ms, and represented a region of reduced amplitude in the Mismatch condition relative to the Match condition. Panel ***B*** (middle row) shows the single significant cluster of relative amplitude difference between the Passive and Mismatch conditions. This cluster extended from ∼30 to 50 Hz, was apparent from ∼25 to 90 ms post the onset of the audible syllable, and manifested as a region of reduced amplitude in the Mismatch condition relative to the Passive condition.

Figure 3*A* shows the spectral power from 30 to 100 Hz for each of the three conditions (Match, Mismatch, and Passive), collapsed across the 12 electrodes-of-interest. The time–frequency plots were baseline corrected using a decibel transform and time locked to the onset of the audible syllable. The units are percentage change in spectral power, relative to the baseline period.

##### Match versus Mismatch

As shown in Figure 3*B* (top row), after controlling for multiple comparisons with permutation-based cluster analysis, a single gamma-band cluster was found to differ in power between the Match and Mismatch conditions (*t*-max_(128)_ = 4.08, *p* = 0.0365, latency-max = 183 ms, frequency-max = 49.9 Hz, electrode-at-max = FC1). Figure 3*B* shows the cluster when collapsing across the 12 electrodes-of-interest. The gamma-band cluster—which lasted from ∼170–220 ms in the frequencies ∼40–65 Hz—reflected a time–frequency region where spectral power was reduced relative to baseline in Match condition, but not reduced relative to baseline in the Mismatch condition. In other words, this reflected a region of increased spectral power in the Mismatch condition, compared with the Match condition.

##### Passive versus Mismatch

As shown in Figure 3*B* (middle row), after controlling for multiple comparisons with permutation-based cluster analysis, a single gamma-band cluster was found to differ in power between the Passive and Mismatch conditions (*t*-max = 4.81, *p* = 0.0041, latency-max = 64 ms, frequency-max = 34 Hz, electrode-at-max = C2). Figure 3*B* shows the cluster when collapsing across the 12 electrodes-of-interest. The gamma-band cluster was observed over a long duration from ∼25 to 90 ms in the frequencies ∼30–50 Hz and reflected a region of increased spectral power in the Passive condition, compared with that in the Mismatch condition.

##### Passive versus Match

After controlling for multiple comparisons, there were no time–frequency clusters that significantly differed in power between the Passive and Match conditions.

#### Lower-frequency bands (5–30 Hz)

**Figure 4.**
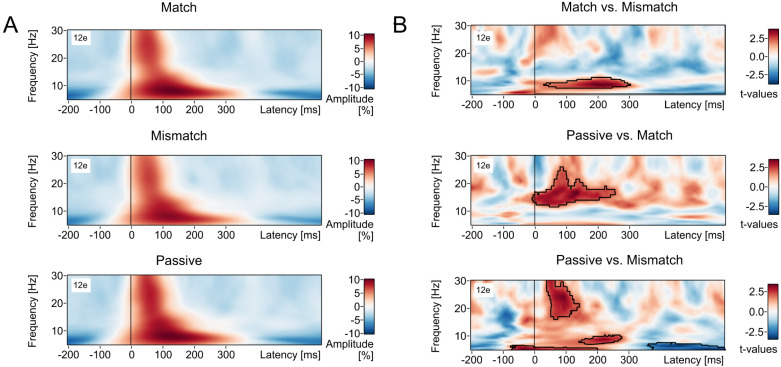
The results of the time–frequency analyses for the lower (i.e., sub-gamma) frequencies (5–30 Hz) for the *n* = 129 participants. Panel ***A*** shows the spectral power from 5 to 30 Hz for each of the three conditions: Match (top row), Mismatch (middle row), and Passive (bottom row). The time–frequency plots are shown collapsed across the 12 electrodes-of-interest (i.e., Cz, C1, C2, FCz, FC1, FC2, CPz, CP1, CP2, Fz, F1, F2), time locked to the onset of the audible syllable, and baseline corrected using a decibel transform in the window −200 to 0 ms. The units are the percent change in amplitude relative to the baseline period. The results of the statistical contrasts are shown in panel ***B***; the units are *t* values. Nonparametric permutation testing (10,000 permutations) was performed on the basis of the paired-samples *t* test (2-tailed). Only those clusters with a cluster value (sum of *t* values of all time–frequency points in the cluster) exceeding 95% of all clusters generated by the permutation procedure were considered statistically significant. Panel ***B*** (top row) shows the single significant cluster of relative amplitude difference between the Match and Mismatch conditions. This cluster, outlined in Panel ***B***, extended across the theta and alpha bands, with a frequency extent of ∼6–10 Hz, and a temporal extent of ∼50–300 ms post-audible-syllable. It represented a region in time–frequency space where the evoked power in the Match condition was greater than in the Mismatch condition. Panel ***B*** (middle row) shows the single cluster of voxels where the evoked power in the Passive condition was greater than in the Match condition. This cluster extended across the alpha and beta bands, with a frequency extent of ∼10–25 Hz, and a temporal extent of ∼0–250 ms post-audible-syllable. Panel ***B*** (bottom row) shows the two clusters that were found to differ in power between the Passive and Mismatch conditions. The first cluster was a region in time–frequency space where the evoked power in the Passive condition was greater than in the Mismatch condition. [Note that this was a single cluster when the 12 electrodes-of-interest were considered separately (as they were for the statistical analysis) but appeared as three separate clusters when collapsing across this set of electrodes, as they were in this figure.] This cluster extended across the theta, alpha, and beta bands, and extended until ∼350 ms post-audible-syllable. The second cluster, in contrast, was a region where evoked power in the Passive condition was reduced relative to the Mismatch condition. This cluster occurred in the theta band, with a frequency extent of ∼5–7 Hz, and a temporal extent of ∼350–600 ms post-audible-stimulus.

Figure 4 shows the spectral power from 5 to 30 Hz for each of the three conditions (Match, Mismatch, and Passive). Data are shown collapsed across the 12 electrodes-of-interest. The time–frequency plots were baseline corrected (from −200 to 0 ms) using a decibel transform and time locked to the onset of the audible syllable. The units are percentage change in spectral power, relative to the baseline period.

##### Match versus Mismatch

As shown in Figure 4*B* (top row), after controlling for multiple comparisons with permutation-based cluster analysis, a single cluster was found to differ in power between the Match and Mismatch conditions. This cluster was a collection of voxels where the evoked power in the Match condition was greater than that in the Mismatch condition. This cluster occurred across the theta and alpha bands, with a frequency extent of ∼6–10 Hz and a temporal extent of ∼50–300 ms post-audible-syllable. The specifications for this cluster were as follows: *t*-max_(128)_ = 4.11, *p* < 0.00780, latency-max = 198 ms, frequency-max = 8.7 Hz, electrode-at-max = Cz.

##### Passive versus Match

As shown in Figure 4*B* (middle row), after controlling for multiple comparisons, a single large cluster was found to differ in power between the Passive and Match conditions. This cluster was a collection of voxels where the evoked power in the Passive condition was greater than in the Match condition. This cluster occurred across the alpha and beta bands, within a frequency extent of ∼10–25 Hz, and a temporal extent of ∼0–250 ms post-audible-syllable. The specifications for this cluster were as follows: *t*-max_(128)_ = 4.06, *p* < 0.00390, latency-max = 76 ms, frequency-max = 17.2 Hz, electrode-at-max = CPz.

##### Passive versus Mismatch

As shown in Figure 4*B* (bottom row), after controlling for multiple comparisons, there were two clusters that were found to differ in power between the Passive and Mismatch conditions. The first cluster was a collection of voxels where the evoked power in the Passive condition was greater than in the Mismatch condition. [Note that this was a single cluster when the 12 electrodes-of-interest were considered separately (as they were for the statistical analysis) but appeared as three separate clusters when collapsing across this set of electrodes, as they were in Fig. 4*B*.] This cluster extended across the theta, alpha, and beta bands and extended until ∼350 ms post-audible-syllable. The specifications for this cluster were as follows: *t*-max_(128)_ = 3.67, *p* < 0.00290, latency-max = 197 ms, frequency-max = 8.7 Hz, electrode-at-max = Cz.

The second cluster was a collection of voxels where the evoked power in the Passive condition was less than the Mismatch condition. This cluster occurred across the theta band, with a frequency extent of ∼5–7 Hz, and a temporal extent of ∼350–600 ms post-audible-stimulus. The specifications for this Mismatch > Passive cluster were as follows: *t*-max_(128)_ = 4.17, *p* < 0.00490, latency-max = 461 ms, frequency-max = 6.4 Hz, electrode-at-max = F2.

## Discussion

There were four key findings in the present study. In regard to the ERP data, (1) the production of an inner syllable reduced the amplitude of the N1 component of the auditory evoked potential elicited by a simultaneously presented audible syllable, but only if the inner and audible syllables were matched on content, i.e., only in the Match condition, relative to both the Passive and Mismatch conditions. Conversely, (2) the amplitude of the P2 component of the auditory evoked potential was reduced only if the inner and audible syllables differed in content, i.e., only in the Mismatch condition, relative to the Passive and Match conditions. In regard to the time–frequency data, (3) after controlling for multiple comparisons using a cluster-based permutation approach, there was a single time–frequency region that differed in spectral power between the Match and Mismatch conditions. This oscillation extended from ∼40 to 65 Hz, was apparent from ∼170 to 220 ms, and represented a region of increased relative power in the Mismatch condition relative to the Match condition. Finally, (4) there was a temporally broad region of power reduction in the Match condition relative to the Mismatch condition in a cluster which extended across the theta and alpha bands, within a frequency extent of ∼6–10 Hz and a temporal extent of ∼50–300 ms post-audible-syllable.

The patterns observed in the ERP data provide a near-perfect replication of the ERP results reported in our previous study (Whitford et al., 2017), which used an identical experimental design but with less statistical power (Hall et al., 2023) due to its substantially smaller participant sample (*n* = 42 participants, with no overlap with the present study). In terms of the N1, the finding that N1 suppression was maximal in the Match condition is reminiscent of the phenomenon of SIS in the overt speech literature, in which N1 suppression to a vocalized sound is maximal when the expected auditory properties of a sound matches its actual auditory properties (Behroozmand et al., 2016). Given that the phenomenon of SIS is widely believed to reflect the action of a corollary discharge (Crapse and Sommer, 2008; Niziolek et al., 2013), the similarity between our results and those reported in the SIS literature suggests that the inner speech may also elicit a precise, content-specific efference copy, as opposed to a generic, nonspecific neural prediction. Ultimately, these results are consistent with the idea that inner speech is a “kind of action” that is closely related to overt speech (Jones and Fernyhough, 2007). The consistency of the current results with our previous work provides evidence of the reliability of the “inner SIS” effect. Such reliability is essential if the phenomenon is to have utility as a potential biomarker for inner speech integrity. The only slight point of difference with Whitford et al. (2017) was that there was a hint of a reduction in the N1 amplitude in the Mismatch condition compared with the Passive condition in the present study. While this difference did not reach statistical significance, it did trend in that direction and thus may provide at least partial support for the prediction that if inner speech elicits a corollary discharge with detailed auditory properties, then N1 amplitude should be reduced in Mismatch compared with Passive, as there is presumably a greater degree of overlap in the auditory properties between /BA/ and /BI/ in the Mismatch condition than between /BA/ and nothing in the Passive condition (Patel, 2022).

In terms of the P2 component, P2 amplitude was reduced in the Mismatch condition (with large effect size), relative to both the Match and Passive conditions, again replicating the results of Whitford et al. (2017). In contrast to the N1 component, whose functional significance is believed to involve low-level “sensory predictions” about the sensory consequences of self-generated sounds, ostensibly via corollary discharge-related mechanisms (Knolle et al., 2019), the functional significance of the auditory P2 is less clear and remains a matter of debate in the literature. However, there is growing evidence that the P2 may reflect something more like “cognitive predictions”, or the “conscious evaluation of action outcomes” (Crowley and Colrain, 2004; Stekelenburg and Vroomen, 2012; Knolle et al., 2019; Korka et al., 2022). Consistent with this idea, it has been suggested that, in the context of sensory attenuation: “whereas the N1-suppression may reflect the unconscious, automatic formation of a prediction, preparing the auditory cortex to receive sensory input, the P2-reduction may reveal a later, more conscious processing stage of the generation of a prediction” (Knolle et al., 2019). In this framework, the P2 amplitude reductions observed in the Mismatch condition in the present study might reflect participants consciously recognizing that the difference in content between the inner and audible syllable. In other words, the P2 reduction may reflect a cognitive expectancy violation, in contrast to the more low-level, “sensory” prediction indexed by the N1. This idea that P2 amplitude reductions could index “cognitive” prediction errors is also consistent with previous studies which have found that mispredicted auditory stimuli elicit smaller P2 amplitudes compared with expected auditory stimuli (Chung et al., 2022; Hsu et al., 2015).

In terms of the oscillations data, there is some existing evidence suggesting that the properties of inner speech are indexed through neural oscillations. For example, Li et al. (2020) found that the temporal properties of inner speech (specifically, the rhythm of imagined singing) was associated with changes in phase coherence in those frequencies that matched the rhythm of the song, while Moon et al. (2022) found the covert speech was preferentially associated with increases in low-gamma and beta-band power, relative to lower-frequency bands (e.g., delta, theta, alpha; Li et al., 2020; Moon et al., 2022). In terms of the present study, there was a single region of difference in gamma-band power between the Match and Mismatch conditions. This region of difference, which reflected increased power in the Mismatch condition, occurred ∼170–220 ms and thus began within, but extended beyond, the P2 time window. In keeping with the above distinction between “sensory” and “cognitive” predictions, the relatively late time course of this gamma-band oscillation may suggest that it may represent a “cognitive prediction error” regarding the expected content of the audible syllable. This possibility, while speculative, is consistent with existing evidence that gamma oscillations may be involved in predictive coding. Specifically, it has been suggested that gamma oscillations can reflect a prediction error signal that is generated when feedforward information in sensory cortex does not match top-down predictions arriving from higher-order areas that are reflected in alpha/beta oscillations (Wang, 2010; Arnal et al., 2011; Arnal and Giraud, 2012). Therefore, gamma oscillations, derived from time–frequency analysis, can provide greater insight into the neural mechanisms underlying inner speech processing than ERPs alone, as the network mechanisms underlying gamma oscillations in predictive coding are better understood than those mechanisms manifested as ERPs.

Contrary to our hypothesis, there was no difference in gamma-band activity in the N1 time window between the Match and Mismatch conditions, in spite of the highly significant differences in N1 amplitude. This result suggests that the corollary discharge that we, and others, have suggested is generated by inner speech may not be associated with a discrete gamma-band oscillation (Tian and Poeppel, 2010; Whitford et al., 2017; Jack et al., 2019), assuming that the N1 component is preferentially affected by the corollary discharge generated by inner speech.

In addition to the gamma-band oscillation around the P2 window, there was also a significant reduction in gamma-band power in the Mismatch condition relative to the Passive condition from ∼30–50 Hz and 25–90 ms. While the underlying cause of this oscillation is not clear, it is notable that numerous electrocorticographic (Crone et al., 2001; Flinker et al., 2010; Fukuda et al., 2010; Greenlee et al., 2011) and electroencephalographic (Ford et al., 2002) studies have found overt speech to be associated with transient increases in gamma-band power, compared with passive listening. This suggests that, whatever the cause of this oscillation may be, it is unlikely to be caused by artifactual micromovements of the articulator organs that have been argued to co-occur with inner speech (Nalborczyk et al., 2020).

In regard to the time–frequency analysis of the lower (i.e., sub-gamma) frequency bands, as shown in Figure 4, the two main findings were the following: (1) the audible phoneme evoked significantly more power in the Match condition than that in the Mismatch condition, in a cluster that extended across the theta and alpha bands, within a frequency extent of ∼6–10 Hz and a temporal extent of ∼50–300 ms post-audible-syllable; and (2) the audible phoneme evoked significantly more power in the Passive condition, relative to both the Match and Mismatch conditions, in several clusters across theta, alpha, and beta bands, from the period around syllable onset until ∼300 ms post-audible-syllable. Together these findings suggest that the lower (i.e., sub-gamma) frequency bands are affected by inner speech production, and the difference between the Match and Mismatch conditions may suggest that these lower frequencies—particularly theta and alpha—are sensitive to inner speech content. While this is a potentially significant finding, its theoretical implications are challenging to unpack in the context of the existing literature. For example, the study of Yao et al. (2021) found that more “vivid” inner speech (as elicited by silently reading “direct” quotes) was associated with higher activity in the theta band than less “vivid” inner speech (elicited by “indirect” quotes). Given this, one explanation for our findings might be that the vividness of the inner syllable was reduced when it co-occurred with an audible syllable that mismatched on content. However, this account would have difficulty explaining why theta power was also reduced in the Mismatch condition relative to the Passive condition, as the (reduced) vividness of the inner syllable in the Mismatch condition would presumably still be higher than the Passive condition, in which participants did not produce an inner phoneme at all. Another possibility is that the reduction in theta power in the Mismatch condition reflected a generic “expectancy violation” signal caused by the incongruency between the inner and audible syllables. Prediction errors have long been associated with changes in theta-band activity in the context of reward, across time windows that are comparable with those observed in the present study (Cavanagh et al., 2010; Mas-Herrero and Marco-Pallarés, 2014). However, this explanation is inconsistent with the fact that these previous studies have typically found prediction errors to elicit transient increases in theta-band power, as opposed to the reductions we observed here. Another interesting possibility is that the higher theta power observed in the Match and Passive conditions is indicative of a more coherent, integrated phoneme representation, as there is only a single syllable to represent in these two conditions. In contrast, the lower theta power in the Mismatch condition may be indicative of a less integrated representation (potentially reflecting a disruption in oscillatory phase; Yao et al., 2021) due to the presence of the two mismatching syllables. One final possibility is that the observed reductions in theta power in the Mismatch condition, relative to both the Passive and Match conditions, simply reflect the P2 amplitude reductions observed in the Mismatch condition in the ERP data, as opposed to any differences in oscillatory brain activity per se. Elucidating the underlying causes of theta reductions in the Mismatch conditions would be a worthwhile aim for future studies.

With regard to interpreting these results, it should be noted that is difficult to definitively determine on the basis of the current design whether the observed effects are caused by inner speech per se, or a more generic prediction error mechanism that signals a discrepancy between the “expected” and “actual” stimulus. For example, it is possible that the reduced P2 amplitude observed in the Mismatch condition was due to an expectancy violation between what participants expected to hear (e.g., /ba/) and what they actually heard (e.g., /bi/), rather than necessarily being dependent on inner speech production. It is also possible that the observed effects were due to inner speech, but not (as we have suggested) due to suppression of audible phonemes by production of inner speech. For example, an alternative possibility is that the incongruent auditory stimuli interrupted inner speech production more in the Mismatch condition than the Match condition and that it was this disruption that underpinned the observed results. We are currently running experiments which aim to differentiate between these different explanations as they have substantially different theoretical implications.

It is also important to consider the scope of the present findings within the inner speech literature. It should be noted that the “subtype” of inner speech that we investigated in the present study (i.e., the silent articulation of an isolated inner syllable, wilfully produced, at a precisely designated time) is a very specific, and quite esoteric, form of inner speech which does not encompass the many and varied forms of the phenomenon, which includes internal dialogues, spontaneous (i.e., unwilled) inner speech, the mental simulation of other people's voices, etc. It is possible that these different subtypes of inner speech rely on different neural/cognitive mechanisms that may or may not involve silent articulation (Hurlburt et al., 2016; Tian et al., 2016; Alderson-Day et al., 2018; Pratts et al., 2023). It remains to be seen whether the phenomenon of “inner speaking-induced suppression” observed here generalizes to these other, arguably more naturalistic, forms of inner speech.

In conclusion, this study demonstrated that the content of a person's inner speech—what they are saying into their mind's ear—can be differentiated on the basis of its distinctive electrophysiological signature. Specifically, we have demonstrated that it is possible to distinguish between two inner syllables (/BA/ and /BI/) on the basis of their influence on the following: (1) N1 amplitude of the ERP, (2) P2 amplitude of the ERP, (3) gamma-band oscillations, and (4) theta/alpha-band oscillations evoked by a simultaneously presented audible syllable. While the N1 and P2 findings replicate our previously published results (Whitford et al., 2017; Jack et al., 2019), thus demonstrating the reliability of these measures in a large sample (*n* = 129), our finding of increased gamma-band activity and decreased theta/alpha-band activity when the inner and audible syllables were mismatched on content has not (to our knowledge) been reported previously. The gamma-band finding is consistent with the idea that “late” gamma-band activity may index consciously perceived expectancy violations or “cognitive prediction errors.” The ability to determine the content of a person's inner speech on the basis of a noninvasive biometric signal (i.e., scalp-recorded electroencephalography) could have significant commercial and industrial applications, such as in regard to the development of brain–computer interfaces (Nieto et al., 2022). It may also have clinical implications with regard to the treatment and monitoring of auditory-verbal hallucinations, given that these have repeatedly been associated with aberrant neural oscillations in the gamma frequency band (Hirano et al., 2015; Hirano and Uhlhaas, 2021) and have long been conceptualized as arising from dysfunctions in inner speech (Moseley et al., 2013).

## Synthesis

Reviewing Editor: Anne Keitel, University of Dundee Decisions are customarily a result of the Reviewing Editor and the peer reviewers coming together and discussing their recommendations until a consensus is reached. When revisions are invited, a fact-based synthesis statement explaining their decision and outlining what is needed to prepare a revision will be listed below. The following reviewer(s) agreed to reveal their identity: Xing Tian. Note: If this manuscript was transferred from JNeurosci and a decision was made to accept the manuscript without peer review, a brief statement to this effect will instead be what is listed below.

Both reviewers agreed that their comments have been thoroughly addressed and that the manuscript is ready for publication. The decision "Revise - Reviewing editor review only" was reached to allow the authors to implement an interpretation regarding the low and high theta findings, if they wish to do so (see below). Once the manuscript is resubmitted, the editor will swiftly accept the manuscript.

*****

Reviewer #1

Advances the Field (Required)

The paper replicates previous research while additionally revealing new observations regarding theta/alpha and gamma oscillatory signatures associated with inner speaking through added analyses.

Comments to the Authors (Required)

The authors have done a thorough job addressing my comments. Thank you very much for the effort. I am pleased to see the additional analyses reveal interesting, albeit challenging to interpret, effects in the lower theta-alpha frequencies.

Regarding the interpretation of the theta power difference, I wonder if the higher theta power observed in the Match and Passive conditions indicates a more coherent, integrated phoneme representation, as there is only one phoneme to represent. In contrast, the lower theta power in the Mismatch condition may suggest a less integrated and more varied representation, reflecting a disruption in the oscillatory phase due to the presence of two mismatched phonemes?

If the authors or the editor think this could provide a plausible interpretation, it may be beneficial to include it as food for thought for the readers.

*****

Reviewer #2

Advances the Field (Required)

The manuscript provides some advances on the neural indices of mental imagery in the spectral domain.

Comments to the Authors (Required)

The authors have answered all my questions.

## Author Response

6 January 2025 Dear Prof. Keitel, Re. Decision on eNeuro eN-TNWR-0297-24R1 Thank you very much for all your efforts on our manuscript, we very much appreciate them. We are delighted to hear that the manuscript is now acceptable for publication in eNeuro, pending the final minor addition detailed below.

In regards to Reviewer 1's final suggestion: we thank the Reviewer for raising this very interesting and thought-provoking possibility. We have added this possibility to the Discussion section the revised manuscript, and highlighted it in red font.

Another interesting possibility is that the higher theta power observed in the Match and Passive conditions is indicative of a more coherent, integrated phoneme representation, as there is only a single syllable to represent in these two conditions. In contrast, the lower theta power in the Mismatch condition may be indicative of a less integrated representation (potentially reflecting a disruption in oscillatory phase, e.g. Yao et al., 2021) due to the presence of the two mismatching syllables.

If you have any questions regarding the revised submission, please do not hesitate to contact me.

Thank you again for everything you have done, and we very much look forward to publishing in eNeuro again in the future.

Yours sincerely, Thomas Whitford, PhD.

Professor School of Psychology University of New South Wales (UNSW Sydney) Sydney NSW 2052 Australia
